# Editorial for Special Issue “Sample Preparation-Quo Vadis: Current Status of Sample Preparation Approaches-2nd Edition”

**DOI:** 10.3390/molecules27196142

**Published:** 2022-09-20

**Authors:** Victoria Samanidou, Irene Panderi

**Affiliations:** 1 Laboratory of Analytical Chemistry, Department of Chemistry, Aristotle University of Thessaloniki, GR-54124 Thessaloniki, Greece; 2 Laboratory of Pharmaceutical Analysis, Division of Pharmaceutical Chemistry, Faculty of Pharmacy, National and Kapodistrtian University of Athens Panepistimiopolis-Zografou, GR 15771 Athens, Greece

Sample preparation is and will always be the most important step in chemical analysis. Numerous techniques, methods, methodologies and approaches are published in the literature offering a wide range of analytical tools to the lab practitioner.

Analytical scientists all over the world try to develop protocols for a plethora of analytes in various sample matrices.

In the last decade, sample pre-treatment advances followed green chemistry and green analytical chemistry demands, focusing on miniaturization and automation, using the lowest possible amounts of organic solvents.

The question is how far we have gone until now as well as what the future perspectives are.

To answer this question, analytical chemists were invited to share their experience in the field and report on the recent advances in sample preparation approaches.

Eleven excellent manuscripts were the outcome of our invitation including four review articles and seven original research articles in the first edition of the Special Issue “Sample Preparation-Quo Vadis: Current Status of Sample Preparation Approaches”.

The second edition is a collection of ten significant contributions to the field of sample preparation. It includes two highly interesting and comprehensive review articles and eight innovative research articles.

José S. Câmara et al. present a comprehensive overview on the Green Extraction Techniques as Advanced Sample Preparation Approaches in Biological, Food, and Environmental Matrices (https://www.mdpi.com/1420-3049/27/9/2953 (accessed on 31 July 2022)).

Aliya Nur Hasanah et al. report on the Factors Affecting Preparation of Molecularly Imprinted Polymer and Methods on Finding Template-Monomer Interaction as the Key of Selective Properties of the Materials (https://www.mdpi.com/1420-3049/26/18/5612 (accessed on 31 July 2022)).

A feature paper on the Multi-Element Analysis Based on an Automated On-Line Microcolumn Separation/Preconcentration System Using a Novel Sol–Gel Thiocyanatopropyl-Functionalized Silica Sorbent Prior to ICP-AES for Environmental Water Samples is presented by Natalia Manousi et al. (https://www.mdpi.com/1420-3049/26/15/4461 (accessed on 31 July 2022)).

Microwave-Assisted Extraction Coupled to HPLC-UV Combined with Chemometrics for the Determination of Bioactive Compounds in Pistachio Nuts and the Guarantee of Quality and Authenticity is proposed by Natasa P. Kalogiouri et al. (https://www.mdpi.com/1420-3049/27/4/1435 (accessed on 31 July 2022)).

Mariana N. Oliveira et al. propose the Application of Bar Adsorptive Microextraction for the Determination of Levels of Tricyclic Antidepressants in Urine Samples (https://www.mdpi.com/1420-3049/26/11/3101 (accessed on 31 July 2022)).

Micro Salting-Out Assisted Matrix Solid-Phase Dispersion: A Simple and Fast Sample Preparation Method for the Analysis of Bisphenol Contaminants in Bee Pollen is presented by Jianing Zhang et al. (https://www.mdpi.com/1420-3049/26/8/2350 (accessed on 31 July 2022)).

A Simple and Reliable Dispersive Liquid-Liquid Microextraction with Smartphone-Based Digital Images for Determination of Carbaryl Residues in *Andrographis paniculata* Herbal Medicines Using Simple Peroxidase Extract from *Senna siamea* Lam. Bark is proposed by Sam-ang Supharoek et al. (https://www.mdpi.com/1420-3049/27/10/3261 (accessed on 31 July 2022)).

Maria S. Synaridou et al. present a Fluorimetric Analytical method for the determination of Five Amino Acids in Chocolate (https://www.mdpi.com/1420-3049/26/14/4325 (accessed on 31 July 2022)).

Stefani Fertaki et al. report on the Measuring Bismuth Oxide Particle Size and Morphology in Film-Coated Tablets (https://www.mdpi.com/1420-3049/27/8/2602 (accessed on 31 July 2022)).

Panagiota Papaspyridakou et al. report a Comparative Study of Sample Carriers for the Identification of Volatile Compounds in Biological Fluids Using Raman Spectroscopy (https://www.mdpi.com/1420-3049/27/10/3279 (accessed on 31 July 2022)).

The Guest Editors wish to thank all authors for their fine contribution and invite analytical chemists to submit their relative work to the third edition until the 31st of January 2023. For more information, the readers are advised to visit the site: https://susy.mdpi.com/academic-editor/special_issues/process/1103908.

